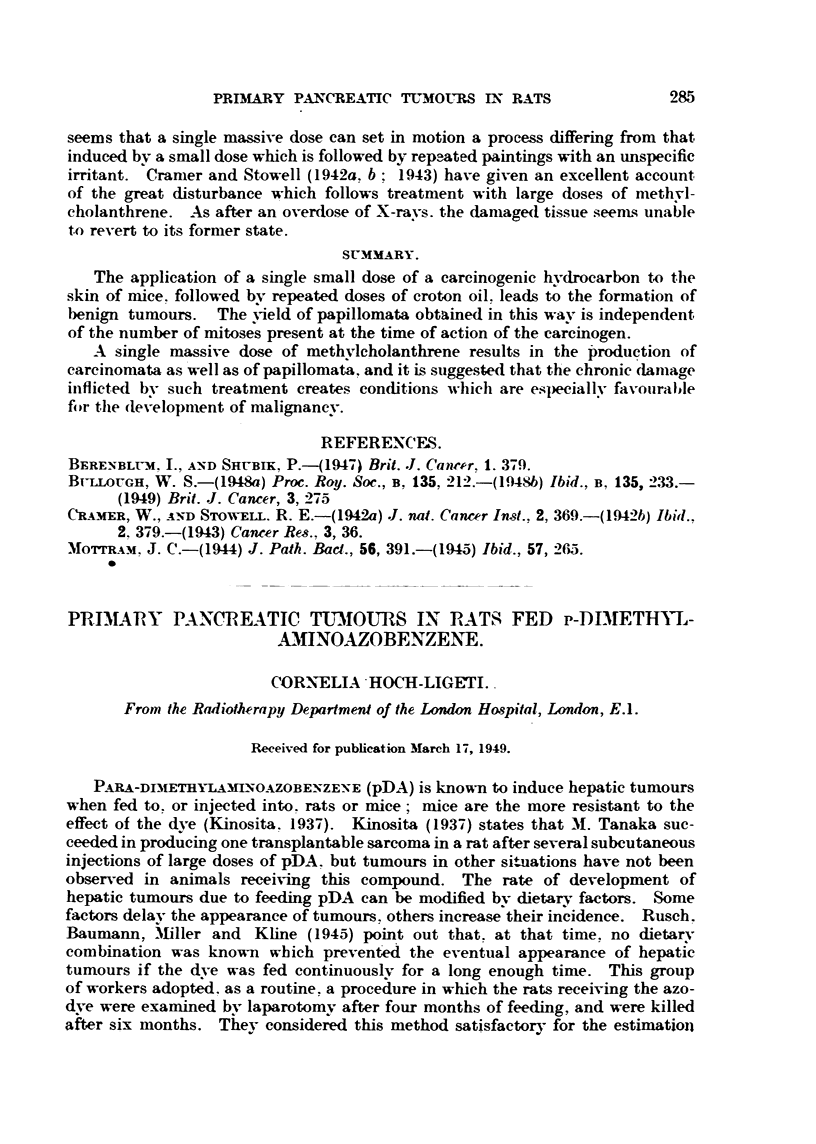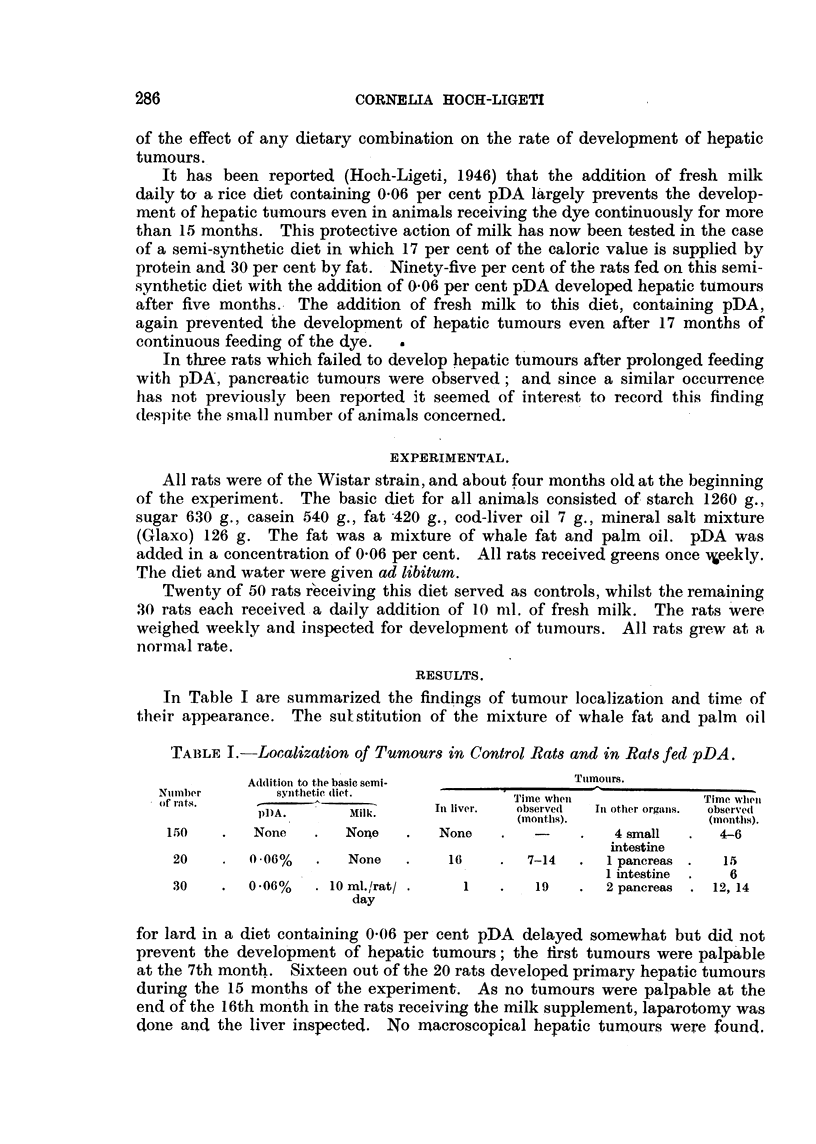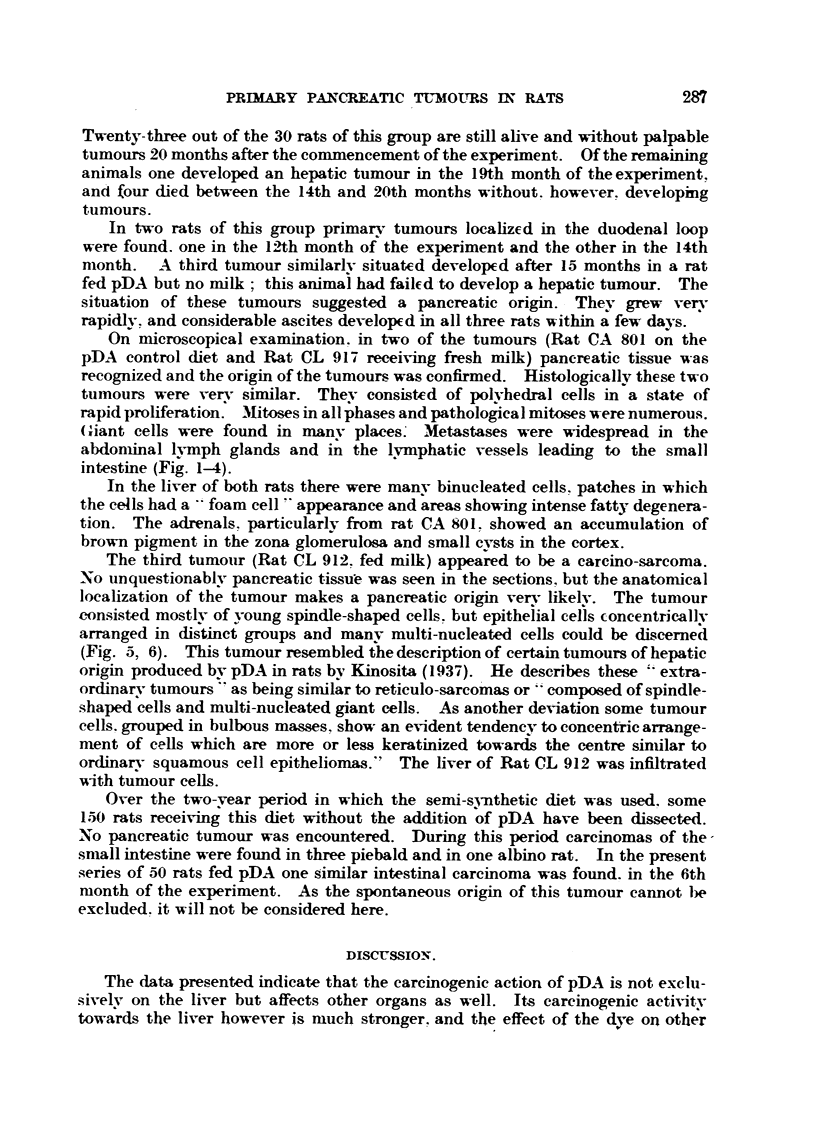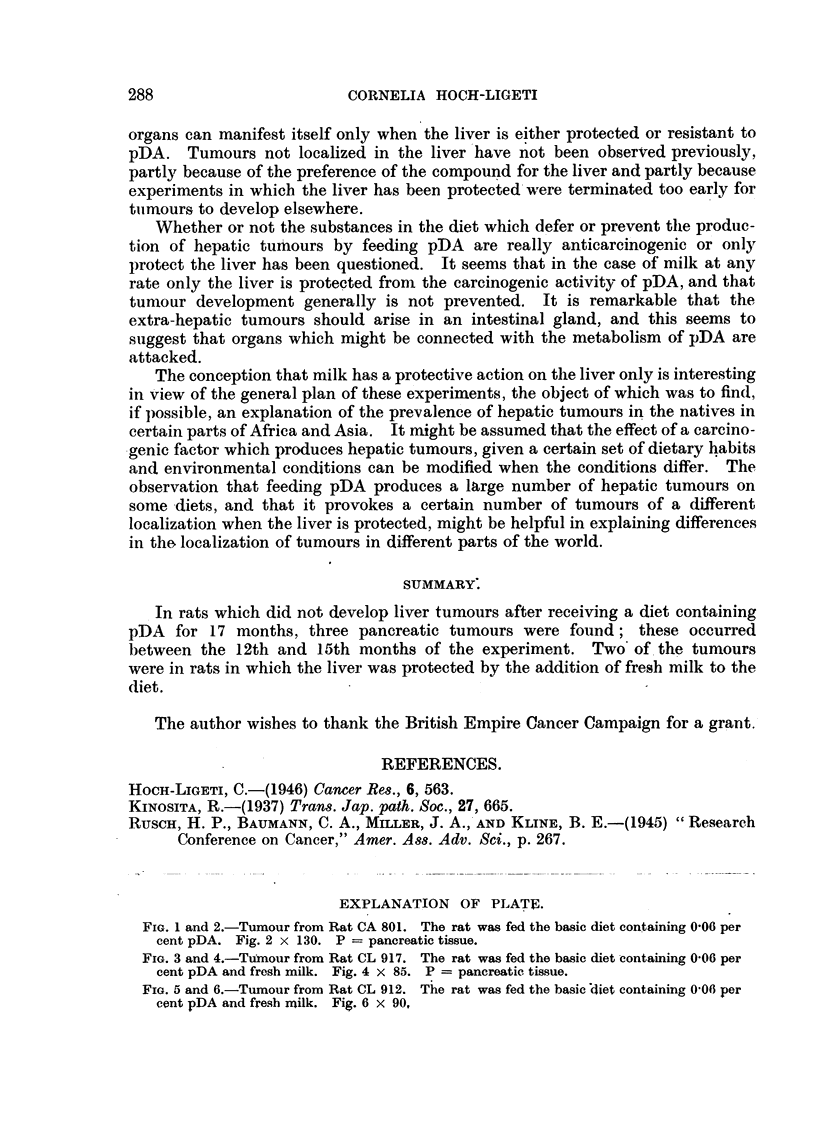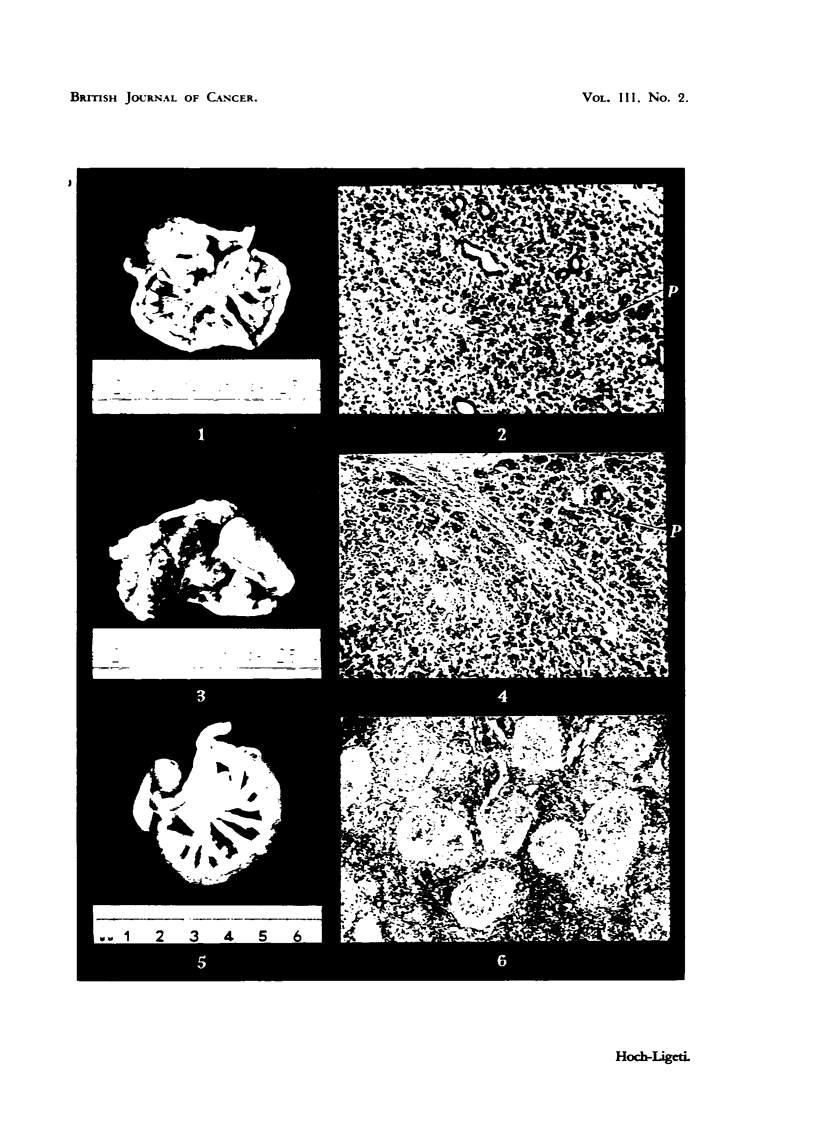# Primary Pancreatic Tumours in Rats Fed P-Dimethylaminoazobenzene

**DOI:** 10.1038/bjc.1949.33

**Published:** 1949-06

**Authors:** Cornelia Hoch-Ligeti

## Abstract

**Images:**


### PRIMARY PANCREATIC TUMOURS IN RATS FED p-DIMETHYLAMINOAZOBENZENE.

CORNELIA HOCH-LIGETI.

*From the Radiotherapy Department of the London Hospital, London, E.1.*

Received for publication March 17, 1949.

Para-dimethylaminoazobenzene (pDA) is known to induce hepatic tumours when fed to, or injected into, rats or mice ; mice are the more resistant to the effect of the dye (Kinosita, 1937). Kinosita (1937) states that M. Tanaka succeeded in producing one transplantable sarcoma in a rat after several subcutaneous injections of large doses of pDA, but tumours in other situations have not been observed in animals receiving this compound. The rate of development of hepatic tumours due to feeding pDA can be modified by dietary factors. Some factors delay the appearance of tumours, others increase their incidence. Rusch, Baumann, Miller and Kline (1945) point out that, at that time, no dietary combination was known which prevented the eventual appearance of hepatic tumours if the dye was fed continuously for a long enough time. This group of workers adopted, as a routine, a procedure in which the rats receiving the azo-dye were examined by laparotomy after four months of feeding, and were killed after six months. They considered this method satisfactory for the estimation of the effect of any dietary combination on the rate of development of hepatic tumours.

It has been reported (Hoch-Ligeti, 1946) that the addition of fresh milk daily to a rice diet containing 0·06 per cent pDA largely prevents the development of hepatic tumours even in animals receiving the dye continuously for more than 15 months. This protective action of milk has now been tested in the case of a semi-synthetic diet in which 17 per cent of the caloric value is supplied by protein and 30 per cent by fat. Ninety-five per cent of the rats fed on this semi-synthetic diet with the addition of 0·06 per cent pDA developed hepatic tumours after five months. The addition of fresh milk to this diet, containing pDA, again prevented the development of hepatic tumours even after 17 months of continuous feeding of the dye.

In three rats which failed to develop hepatic tumours after prolonged feeding with pDA, pancreatic tumours were observed; and since a similar occurrence has not previously been reported it seemed of interest to record this finding despite the small number of animals concerned.

### EXPERIMENTAL.

All rats were of the Wistar strain, and about four months old at the beginning of the experiment. The basic diet for all animals consisted of starch 1260 g., sugar 630 g., casein 540 g., fat 420 g., cod-liver oil 7 g., mineral salt mixture (Glaxo) 126 g. The fat was a mixture of whale fat and palm oil. pDA was added in a concentration of 0·06 per cent. All rats received greens once weekly. The diet and water were given *ad libitum.*

Twenty of 50 rats receiving this diet served as controls, whilst the remaining 30 rats each received a daily addition of 10 ml. of fresh milk. The rats were weighed weekly and inspected for development of tumours. All rats grew at a normal rate.

### RESULTS.

In Table I are summarized the findings of tumour localization and time of their appearance. The substitution of the mixture of whale fat and palm oil

TABLE I.—*Localization of Tumours in Control Rats and in Rats fed pDA.*

| Number of rats. | Addition to the basic semi-synthetic diet. | | Tumours. | | | |
|---|---|---|---|---|---|---|
| | pDA. | Milk. | In liver. | Time when observed (months). | In other organs. | Time when observed (months). |
| 150 | None | None | None | — | 4 small intestine | 4–6 |
| 20 | 0·06% | None | 16 | 7–14 | 1 pancreas<br>1 intestine | 15<br>6 |
| 30 | 0·06% | 10 ml./rat/day | 1 | 19 | 2 pancreas | 12, 14 |

for lard in a diet containing 0·06 per cent pDA delayed somewhat but did not prevent the development of hepatic tumours; the first tumours were palpable at the 7th month. Sixteen out of the 20 rats developed primary hepatic tumours during the 15 months of the experiment. As no tumours were palpable at the end of the 16th month in the rats receiving the milk supplement, laparotomy was done and the liver inspected. No macroscopical hepatic tumours were found.

Twenty-three out of the 30 rats of this group are still alive and without palpable tumours 20 months after the commencement of the experiment. Of the remaining animals one developed an hepatic tumour in the 19th month of the experiment, and four died between the 14th and 20th months without, however, developing tumours.

In two rats of this group primary tumours localized in the duodenal loop were found, one in the 12th month of the experiment and the other in the 14th month. A third tumour similarly situated developed after 15 months in a rat fed pDA but no milk ; this animal had failed to develop a hepatic tumour. The situation of these tumours suggested a pancreatic origin. They grew very rapidly, and considerable ascites developed in all three rats within a few days.

On microscopical examination, in two of the tumours (Rat CA 801 on the pDA control diet and Rat CL 917 receiving fresh milk) pancreatic tissue was recognized and the origin of the tumours was confirmed. Histologically these two tumours were very similar. They consisted of polyhedral cells in a state of rapid proliferation. Mitoses in all phases and pathological mitoses were numerous. Giant cells were found in many places. Metastases were widespread in the abdominal lymph glands and in the lymphatic vessels leading to the small intestine (Fig. 1–4).

In the liver of both rats there were many binucleated cells, patches in which the cells had a " foam cell " appearance and areas showing intense fatty degeneration. The adrenals, particularly from rat CA 801, showed an accumulation of brown pigment in the zona glomerulosa and small cysts in the cortex.

The third tumour (Rat CL 912, fed milk) appeared to be a carcino-sarcoma. No unquestionably pancreatic tissue was seen in the sections, but the anatomical localization of the tumour makes a pancreatic origin very likely. The tumour consisted mostly of young spindle-shaped cells, but epithelial cells concentrically arranged in distinct groups and many multi-nucleated cells could be discerned (Fig. 5, 6). This tumour resembled the description of certain tumours of hepatic origin produced by pDA in rats by Kinosita (1937). He describes these " extraordinary tumours " as being similar to reticulo-sarcomas or " composed of spindle-shaped cells and multi-nucleated giant cells. As another deviation some tumour cells, grouped in bulbous masses, show an evident tendency to concentric arrangement of cells which are more or less keratinized towards the centre similar to ordinary squamous cell epitheliomas." The liver of Rat CL 912 was infiltrated with tumour cells.

Over the two-year period in which the semi-synthetic diet was used, some 150 rats receiving this diet without the addition of pDA have been dissected. No pancreatic tumour was encountered. During this period carcinomas of the small intestine were found in three piebald and in one albino rat. In the present series of 50 rats fed pDA one similar intestinal carcinoma was found, in the 6th month of the experiment. As the spontaneous origin of this tumour cannot be excluded, it will not be considered here.

### DISCUSSION.

The data presented indicate that the carcinogenic action of pDA is not exclusively on the liver but affects other organs as well. Its carcinogenic activity towards the liver however is much stronger, and the effect of the dye on other organs can manifest itself only when the liver is either protected or resistant to pDA. Tumours not localized in the liver have not been observed previously, partly because of the preference of the compound for the liver and partly because experiments in which the liver has been protected were terminated too early for tumours to develop elsewhere.

Whether or not the substances in the diet which defer or prevent the production of hepatic tumours by feeding pDA are really anticarcinogenic or only protect the liver has been questioned. It seems that in the case of milk at any rate only the liver is protected from the carcinogenic activity of pDA, and that tumour development generally is not prevented. It is remarkable that the extra-hepatic tumours should arise in an intestinal gland, and this seems to suggest that organs which might be connected with the metabolism of pDA are attacked.

The conception that milk has a protective action on the liver only is interesting in view of the general plan of these experiments, the object of which was to find, if possible, an explanation of the prevalence of hepatic tumours in the natives in certain parts of Africa and Asia. It might be assumed that the effect of a carcinogenic factor which produces hepatic tumours, given a certain set of dietary habits and environmental conditions can be modified when the conditions differ. The observation that feeding pDA produces a large number of hepatic tumours on some diets, and that it provokes a certain number of tumours of a different localization when the liver is protected, might be helpful in explaining differences in the localization of tumours in different parts of the world.

### SUMMARY.

In rats which did not develop liver tumours after receiving a diet containing pDA for 17 months, three pancreatic tumours were found; these occurred between the 12th and 15th months of the experiment. Two of the tumours were in rats in which the liver was protected by the addition of fresh milk to the diet.

The author wishes to thank the British Empire Cancer Campaign for a grant.

### REFERENCES.

HOCH-LIGETI, C.—(1946) *Cancer Res.*, **6**, 563.

KINOSITA, R.—(1937) *Trans. Jap. path. Soc.*, **27**, 665.

RUSCH, H. P., BAUMANN, C. A., MILLER, J. A., AND KLINE, B. E.—(1945) "Research Conference on Cancer," *Amer. Ass. Adv. Sci.*, p. 267.

---

### EXPLANATION OF PLATE.

FIG. 1 and 2.—Tumour from Rat CA 801. The rat was fed the basic diet containing 0·06 per cent pDA. Fig. 2 × 130. P = pancreatic tissue.

FIG. 3 and 4.—Tumour from Rat CL 917. The rat was fed the basic diet containing 0·06 per cent pDA and fresh milk. Fig. 4 × 85. P = pancreatic tissue.

FIG. 5 and 6.—Tumour from Rat CL 912. The rat was fed the basic diet containing 0·06 per cent pDA and fresh milk. Fig. 6 × 90.